# New Appliances and Things Medical

**Published:** 1910-08-13

**Authors:** 


### NEW APPLIANCES AND THINGS MEDICAL.

**[We shall be glad to receive at our Office, 28 & 29 Southampton Street, Strand, London, W.C., from the manufacturers, specimens of all new preparations and appliances.]**

### "COFECTANT" BRAND DISINFECTANTS
(Manufactured by Edward Cook and Co., Ltd., Bow, London, E.).

Specimens of the various products prepared by this well-known firm of sanitary specialists have been submitted to us. The basis of them all is a coal-tar derivative, which is so combined as to serve as an efficient antiseptic foundation for fluid and powdered disinfectants, a surgical antiseptic fluid, two soaps, an ointment, and a preparation for internal administration. All these excellently mounted articles are pervaded in greater or less degree by the by no means unpleasant characteristic smell of the "Cofectant" product. The soaps are first a tar-medicated bath soap, which is pleasant and economical in use and is distinctly a valuable addition to this class of toilet necessary; and, secondly, a more strongly disinfectant soap, which is recommended as a preventive of midge and mosquito bites and, like the bath soap, has the great advantage of being non-poisonous, a noteworthy quality, which applies also to the whole of the "Cofectant" disinfectants. The preparation for internal use consists of a three-minim dose encased in a "membroid"; it may be prescribed with benefit in those cases of extensive fermentation for which thymol, B-naphthol, and similar remedies are often effective. The surgical disinfectant has a very high germicidal co-efficient, and is invisible with hot or cold water in all proportions. The dilutions which experience has dictated are (by volume) as follows: 1 in 300 for disinfecting the site of an operation and for ringworm; 1 in 400 for septic throats, purulent otitis media, etc.; 1 in 500 as a general surgical disinfectant and antiseptic lotion; 1 in 800 for gynæcological and obstetric practice; and weaker still for enemata and antiseptic baths. The "Cofectant" fluid is non-toxic and absolutely safe, besides being a powerful germicide and deodorant; for sinks and drains it is used in a strength of a tablespoonful to a bucket of water; for cesspools and closets of twice that strength; for destroying insect pests in gardens a weaker solution will be found to be efficient. Lastly, the powdered product is a germicide, whose strength is estimated to be fifteen times that of an ordinary 15 per cent. carbolic-acid powder.

